# Deutschsprachige Versionen des Tinnitus Functional Index: Vergleich der beiden validierten deutschsprachigen Versionen des Tinnitus Functional Index für die Schweiz und für Deutschland

**DOI:** 10.1007/s00106-021-01099-w

**Published:** 2021-08-27

**Authors:** Nicole Peter, Tobias Kleinjung, Ricarda Lippuner, Benjamin Boecking, Petra Brueggemann, Birgit Mazurek

**Affiliations:** 1 Klinik für Ohren‑, Nasen- Hals- und Gesichtschirurgie, UniversitätsSpital Zürich, Universität Zürich, Frauenklinikstraße 24, 8091 Zürich, Schweiz; 2 Tinnituszentrum, Charité – Universitätsmedizin Berlin, Chariteplatz 1, 10117 Berlin, Deutschland

**Keywords:** TFI, Fragebogen, Faktoranalyse, Validierung, Interkulturelle Befragungen, TFI, Questionnaire, Factor analysis, Validation, Cross-cultural survey

## Abstract

**Hintergrund:**

Es existieren zwei deutschsprachige, validierte Versionen des Tinnitus Functional Index (TFI), eine für die Schweiz und eine für Deutschland. Der TFI gilt als möglicher neuer Standard-Fragebogen für die Evaluation eines Tinnitus-Schweregrads und einer Tinnitus-Therapie.

**Ziel der Arbeit:**

In Anbetracht der stattfindenden Standardisierung bei der Tinnitus-Evaluation war es unser Ziel, die beiden TFI-Versionen miteinander zu vergleichen und im deutschsprachigem Raum nur eine TFI-Version zu empfehlen.

**Material und Methoden:**

Die beiden deutschsprachigen TFI-Versionen wurden in einer multizentrischen randomisierten Online-Fragebogenstudie im Cross-over-Design miteinander verglichen.

**Ergebnisse:**

Die Gesamtscores der beiden TFI-Versionen unterschieden sich in der gesamten Population nicht. Bei weiterer Aufschlüsselung in Bezug auf die Population und Reihenfolge der abgegeben TFI-Versionen zeigten sich allerdings teilweise signifikante Unterschiede mit jedoch nur moderaten Effektstärken. Dies deutet darauf hin, dass sich die beiden Versionen leicht unterscheiden, aber trotzdem miteinander vergleichbar sind. Bei der Faktoranalyse konnten bei der TFI-Version für Deutschland in der gesamten Population wie auch für die schweizerische Population 6 Faktoren extrahiert werden. Hingegen konnten bei der deutschen Population in beiden TFI-Versionen und bei der schweizerischen Population in der schweizerischen TFI-Version nur 5 Faktoren extrahiert werden.

**Schlussfolgerung:**

Die beiden deutschsprachigen Versionen des TFI sind gut miteinander vergleichbar. Jedoch spricht die Faktoranalyse eher für die Verwendung der TFI-Version für Deutschland im gesamten deutschsprachigen Raum.

## Abstract (en)

**Background:**

Two validated German-language versions of the Tinnitus Functional Index (TFI) exist, one for Switzerland and one for Germany. The TFI is considered to be a possible new standard questionnaire for evaluation of tinnitus severity and tinnitus treatment.

**Objective:**

Considering the standardization taking place in tinnitus evaluation, our aim was to compare the two German-language TFI versions and to recommend only one TFI version in the German-speaking area.

**Materials and methods:**

The two German-language TFI versions were compared in a multicenter and randomized online questionnaire study with a crossover design.

**Results:**

The total score of the two TFI versions did not differ in the total population. However, when further divided in terms of population and order of presentation of the TFI versions, there were significant differences in some cases, albeit with only moderate effect sizes. This suggests that the two versions are slightly different but still comparable. In factor analysis for the TFI version for Germany, in the entire population as well as in the Swiss population, six factors could be extracted. In contrast, for the German and Swiss TFI versions, only five factors could be extracted in the German population, and for the Swiss TFI version, only five factors in the Swiss population.

**Conclusion:**

The two German-language versions of the TFI are well comparable with each other. However, the factor analysis rather argues for use of the TFI version for Germany in the entire German-speaking region.

Tinnitus ist ein häufiges Symptom, das in manchen Fällen zu deutlicher Beeinträchtigung der Lebensqualität führen kann. Um den Schweregrad von Tinnitus zu bewerten und eine Therapie zu evaluieren, wurde 2012 an der Oregon Health and Science University (OHSU) der Fragebogen Tinnitus Functional Index (TFI) entwickelt [16]. Es existieren zwei deutschsprachige validierte Versionen des TFI, eine für die Schweiz und eine für Deutschland. In Anbetracht der stattfindenden Standardisierung bei der Tinnitus-Evaluation war es unser Ziel, nur eine TFI-Version im deutschsprachigem Raum zu empfehlen.

## Hintergrund und Fragestellung

Tinnitus ist eine akustische Wahrnehmung von Geräuschen bei fehlendem akustischem Stimulus. Chronischer Tinnitus ist ein häufiges Symptom und die Prävalenz eines Ohrgeräusches in Europa liegt bei 15 % [3, 4], wobei ein schwerer Tinnitus in ca. 1 % der Bevölkerung vorkommt [4]. Da der individuelle Leidensdruck durch ein Ohrgeräusch für die Empfehlung einer möglichen Therapie von großer Bedeutung ist, kommen neben der Anamnese Selbsteinschätzungs-Fragebögen zum Einsatz. Hierbei werden verschiedene Aspekte des täglichen Lebens erfragt, wie z. B. Konzentration, Schlaf, emotionaler Stress und Lebensqualität, welche durch den Tinnitus beeinträchtigt werden können. Es existieren bereits verschiedene standardisierte Fragebögen, um den Schweregrad eines Ohrgeräusches zu klassifizieren. Im deutschsprachigem Raum ist der Tinnitus-Fragebogen nach Goebel und Hiller weit verbreitet [9]. In internationaler Hinsicht wird aufgrund seiner Validierung in verschiedensten Sprachen, etwa für Deutsch, Niederländisch oder Koreanisch, das Tinnitus Handicap Inventory (THI) am häufigsten gebraucht [12, 15, 17, 22]. Beide Fragebögen wurden in erster Linie für die Feststellung des Tinnitus-Schweregrads entwickelt, werden aber auch zunehmend für die Evaluation von Tinnitus-Therapien im kurzfristigen Verlauf verwendet [14]. Um dieses Problem zu lösen, unterstützte das Tinnitus Research Consortium (TRC) die Entwicklung und Evaluation eines neuen Tinnitus-Messinstruments mit dem Ziel, die kurzfristigen longitudinalen Verläufe einer Tinnitus-Therapie besser abzubilden [20]. In der Folge wurde 2012 ein neuer Fragebogen, der Tinnitus Functional Index (TFI) entwickelt [16, 20]. Mittels explorativer Faktorenanalyse wurden hierbei die verschiedenen Aspekte, die durch Tinnitus beeinträchtigt werden können, in acht Subskalen (Faktoren) unterteilt: Kognition („cognitive subscale“), Hören („auditory subscale“), Penetranz („intrusive subscale“), Schlaf („sleep subscale“), Lebensqualität („quality of life subscale“), Emotion („emotional subscale“), Kontrolle („sense of control subscale“), Entspannung („relaxation subscale“) [5, 8, 16]. Der Fragebogen wurde bereits in verschiedene Sprachen übersetzt und validiert, beispielsweise Japanisch, Niederländisch und Deutsch [5, 18, 19, 21].

**Figure 1:**
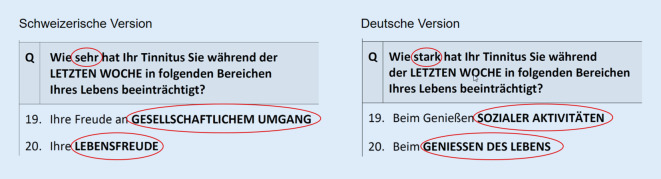

Aufgrund einer separaten Vorwärts-Rückwärts-Übersetzung in Deutschland und in der Schweiz existieren in der deutschen Sprache zwei unterschiedliche TFI-Versionen, die sich in einzelnen Fragen semantisch unterscheiden. Exemplarisch sind die Unterschiede von Frage 19 und 20 in Abb. 1 dargestellt. Die Version für die deutschsprachige Schweiz wurde 2017 validiert [18]. Hierbei konnten jedoch nur 5 der ursprünglichen 8 Faktoren mittels Hauptkomponentenanalyse und Jolliffe-Kriterium (Eigenwerte > 0,7) extrahiert werden [18]. Gleichzeitig wurde auch die TFI-Version für Deutschland validiert [5], wobei die mehrfaktorielle Struktur des Original-TFI [16] mit 8 Faktoren in einer Hauptkomponentenanalyse bestätigt werden konnte. Jedoch wurden die Eigenwerte hier nicht angeben, sodass die deutsche TFI-Version nicht mit der schweizerischen TFI-Version verglichen werden kann.

In Anbetracht der weiten internationaler Verbreitung des TFI-Fragbogens dürfte dieser in Zukunft vermutlich als neuer Goldstandard für die Tinnitus-Bewertung gelten. Angesichts der stattfindenden Standardisierung bei der Tinnitus-Evaluation war es unser Ziel, mittels einer Fragebogenstudie, für die zukünftige Benutzung im deutschsprachigem Raum nur eine deutschsprachige Fragebogen-Version des TFI zu empfehlen. Grundsätzlich besteht hierfür die Möglichkeit, sich auf eine bestehende deutschsprachige TFI-Version zu einigen oder eine neue harmonisierte TFI-Version aus den beiden bestehenden deutschsprachigen TFI-Versionen zu schaffen. Da eine neue harmonisierte TFI-Version eine erneute Validierung nach sich gezogen hätte, schien uns dieses Vorgehen unverhältnismäßig, und aus diesem Grund wurden im Rahmen dieser Studie die beiden deutschsprachigen TFI-Versionen gegenübergestellt. Einerseits wurden die erhobene Gesamtpunktzahl der beiden validierten deutschsprachigen TFI-Versionen miteinander verglichen, andererseits wurden für beide TFI-Versionen Faktoranalysen mit der Frage nach der Anzahl extrahierbarer Faktoren durchgeführt.

## Studiendesign und Untersuchungsmethoden

Es handelte sich um eine randomisierte Online-Fragebogenstudie mit Cross-over-Design. Die Studie wurde einerseits am Tinnituszentrum der Charité und andererseits an der Klinik für Ohren‑, Nasen‑, Hals- und Gesichtschirurgie des UniversitätsSpitals Zürich durchgeführt. Die Studie wurde an beiden Zentren von der zuständigen Ethikkommission bewilligt (EA1/084/19 und BASEC-Nr. 2018-01592). In die Studie wurden Tinnituspatientinnen und -patienten einbezogen, die an den jeweiligen Zentren einen Konsultationstermin vereinbart hatten. Nach Klärung der Ein- und Ausschlusskriterien und Unterzeichnung der Einwilligungserklärung wurden an beiden Zentren die zwei validierten deutschsprachigen TFI-Versionen an alle Probanden abgegeben. Die Reihenfolge der beiden TFI-Versionen wurde nach einer Block-Randomisierung den Probanden zugeteilt, und die Fragebögen wurden online ausgefüllt. Hierbei wurden die beiden TFI-Versionen den Patienten direkt hintereinander dargeboten.

Teilnehmer, die die folgenden Einschlusskriterien erfüllten, wurden in die Studie eingeschlossen: Volljährigkeit (18. Lebensjahr vollendet), vorhandener Tinnitus und ausreichende Deutschkenntnisse. Bei Unmöglichkeit, den Fragebogen aufgrund kognitiver oder körperlicher Einschränkungen auszufüllen, wurde ein Proband ausgeschlossen. Die Patienten wurden aus den laufenden Sprechstunden in den jeweiligen Ländern rekrutiert. Die beiden Populationen waren somit nicht in Bezug auf das Alter oder Geschlecht miteinander abgestimmt. Die Dauer des Ohrgeräusches spielte für die Studienteilnahme keine Rolle.

Gemäß Poweranalyse und Stichprobenumfangsberechnung mussten mindestens 252 Teilnehmer in die Studie eingeschlossen werden.

Die statistische Analyse wurde mithilfe von SPSS (Statistical Packages for Social Sciences, Version 26.0, SPSS Inc., Chicago/IL, USA) durchgeführt.

Die Berechnung der Gesamtpunktzahl erfolgte gemäß dem Urheber Oregon Health and Science University (OHSU) [16]. Alle erhobenen Werte wurden mittels deskriptiver Statistik dargestellt (Mittelwert, Standardabweichung).

Da die Probanden in Deutschland und in der Schweiz beide deutschsprachigen TFI-Versionen in einer vorgängig randomisiert festgelegten Reihenfolge ausfüllten, wurde der Gesamtscore der beiden TFI-Versionen innerhalb derselben Gruppe jeweils mittels eines gepaarten t‑Tests verglichen:

- Gesamtpopulation,
- Land (deutsche und schweizerische Population),
- Reihenfolge der TFI-Versionen bei der gesamten Population,
- Reihenfolge der TFI-Versionen bei der deutschen Population,
- Reihenfolge der TFI-Versionen bei der schweizerischen Population.

Analog dazu wurde mittels eines ungepaarten t‑Tests der Gesamtscore einer TFI-Version zwischen zwei unterschiedlichen Gruppen verglichen.

Bei einem signifikanten Unterschied der Gesamtpunktzahl im t‑Test wurde die Effektstärke gemäß Korrelationskoeffizient *r* berechnet und nach Einteilung von Cohen [7] beurteilt. Zusätzlich wurde bei einem signifikanten Unterschied der Gesamtpunktzahl jede einzelne Frage mittels t‑Test und dazugehöriger Effektstärke verglichen.

Vor Durchführung einer Faktoranalyse bei beiden TFI-Versionen in der gesamten, der deutschen und der schweizerischen Population wurde durch den Bartlett-Test auf Sphärizität und das Kaiser-Meyer-Olkin-Kriterium bestimmt, ob der Datensatz für eine Faktoranalyse geeignet ist [13]. Danach wurde eine Hauptkomponentenanalyse mit orthogonaler Rotationsanalyse durchgeführt, um verschiedene Faktoren zu extrahieren. Um die Anzahl der gültigen Faktoren zu erhalten, wurden die Eigenwerte mittels Jolliffe-Kriterium (Eigenwerte > 0,7) ermittelt [11]. Da in dem Original-TFI [16] eine mehrfaktorielle Struktur mit 8 Faktoren dokumentiert wurde, erfolgte zusätzlich eine Hauptkomponentenanalyse mit orthogonaler Rotationsanalyse mit 8 fixierten Faktoren. Das Signifikanzlevel wurde bei allen Analysen auf *p* ≤ 0,05 festgelegt, falls spezifisch nicht anders angegeben.

## Ergebnisse

Zwischen Oktober 2019 und Juli 2020 nahmen 255 Probanden, davon 128 in Deutschland und 127 in der Schweiz, an der Studie teil. Das mittlere Alter der gesamten Population war 52,2 Jahre (Standardabweichung, SD: 12,0). Das Alter zwischen der deutschen und schweizerischen Population unterschied sich nicht signifikant (*p* = 0,08). Es nahmen weniger Frauen als Männer an der Studie teil (38,8 % Frauen). In Deutschland war der Anteil an Frauen bei 46,1 % höher als in der Schweiz bei 31,5 %. Die Gesamtpunktzahl der TFI-Versionen war zwischen den Geschlechtern in der deutschen Population (in beiden TFI-Versionen *p* = 0,34), sowie in der schweizerischen Population (in beiden TFI-Versionen *p* = 0,55) nicht signifikant unterschiedlich.

Es zeigte sich, dass der gepaarte t‑Test beider TFI-Versionen in der Gesamtpopulation nicht signifikant unterschiedlich war. Interessanterweise zeigte sich, dass die Gesamtpunktzahl bei der gesamten, deutschen und schweizerischen Population jeweils bei der TFI-Version höher war, welche zuerst durchgeführt wurde. Es fand sich ein signifikanter Unterschied bei weiterer Aufschlüsselung in Bezug auf die Reihenfolge der TFI-Abgabe in der gesamten (*p* = 0,000 und *p* = 0,006) und in der schweizerischen Population (*p* = 0,000 und *p* = 0,01). Die Effektstärke des Korrelationskoeffizienten *r *lag hier zwischen 0,24 und 0,49. Gemäß Einteilung von Cohen [7] entspricht ein Wert von *r* = 0,1 einem schwachen Effekt, ein Wert von *r* = 0,3 einem mittleren Effekt und ein Wert von *r* = 0,5 einem starken Effekt. Bei überwiegenden Werten von *r* < 0,5 ist nicht von einem starken Effekt auszugehen. Es zeigte sich allerdings, dass sich die beiden deutschsprachigen TFI-Versionen doch teilweise zu unterscheiden schienen. Weitere gepaarte t‑Tests der einzelnen Fragen erfolgten bei signifikant unterschiedlichen Werten der Gesamtpunktzahl. Hierbei zeigte sich, dass sich insbesondere die Frage 5 zu unterscheiden schien. Die ungepaarten t‑Tests einer TFI-Version zwischen zwei Populationen waren jeweils nicht signifikant.

In Tab. 1 ist eine Übersicht der durchgeführten explorativen Faktoranalysen der gesamten, deutschen und schweizerischen Population abgebildet. Das Maß der Stichprobeneignung nach Kaiser-Meyer-Olkin, die Signifikanz im Bartlett-Test auf Sphärizität und der Bereich der Kommunalitäten zeigten Werte, welche Faktoranalysen in den genannten Populationen erlaubten. In der gesamten Population konnten bei der deutschen TFI-Version mittels Jolliffe-Kriterium 6 Faktoren und bei der schweizerischen TFI-Version 5 Faktoren extrahiert werden. Interessanterweise zeigten sich in der schweizerischen Population bei der deutschen TFI-Version ebenfalls 6 Faktoren, wohingegen bei der schweizerischen TFI-Version nur 5 Faktoren extrahiert werden konnten. In der deutschen Population liessen sich in beiden TFI-Versionen nur jeweils 5 Faktoren extrahieren.

**Table 1:** 

	Gesamte Population		Deutsche Population		Schweizerische Population	
	TFI-CH	TFI-GER	TFI-CH	TFI-GER	TFI-CH	TFI-GER
Anzahl extrahierte Faktoren mittels Jolliffe-Kriterium	5	6	5	5	5	6
Kumulierte Varianz aller Variablen bei o. g. Faktoren (in %)	81,89	84,80	82,73	82,44	82,84	86,10
Erklärung zu den o. g. Faktoren	I & SC, Q & E, R & C	R & SC, Q & E, TFI-22 falsch bei C anstatt Q	R & SC & C, Q & E	E & Q (außer TFI-19 falsch bei A) & C (außer TFI‑7 falsch bei I), SC & R	Q & C & E, I (außer TFI‑2 falsch bei R) & SC	C & R, SC & E
Kumulierte Varianz aller Variablen bei 8 fixierten Faktoren (in %)	88,84	89,20	89,23	89,19	89,57	90,44
Erklärung zu den 8 Faktoren	TFI-22 falsch bei C anstatt Q	TFI-22 falsch bei C anstatt Q	TFI‑9 falsch bei R, TFI-22 falsch bei C	E & Q (außer TFI-19 falsch bei A), TFI‑2 eigener Faktor	SC (außer TFI‑4 eigener Faktor) & E, TFI-20 falsch bei SC & E anstatt Q, TFI-22 falsch bei C anstatt Q	TFI-22 falsch bei C anstatt Q

*TFI* Tinnitus Functional Index, *TFI-CH* schweizerische Version des TFI, *TFI-GER* deutsche Version des TFI, *C* Kognition („cognitive subscale“), *A* Hören („auditory subscale“), *I* Penetranz („intrusive subscale“), *SL* Schlaf („sleep subscale“), *Q* Lebensqualität („quality of life subscale“), *E* Emotion („emotional subscale“), *SC* Kontrolle („sense of control subscale“), *R* Entspannung („relaxation subscale“)

Die Zuteilung der einzelnen Fragen zu den extrahierten Faktoren respektive zu den 8 fixierten Faktoren in Bezug auf die Subskalen der Original-Publikation (C = Kognition, „cognitive subscale“, A = Hören, „auditory subscale“, I = Penetranz, „intrusive subscale“, SL = Schlaf, „sleep subscale“, Q = Lebensqualität, „quality of life subscale“, E = Emotion, „emotional subscale“, SC = Kontrolle, „sense of control subscale“, R = Entspannung, „relaxation subscale“) sind ebenfalls in Tab. 1 ersichtlich. Hierbei konnten die Fragen der deutschen TFI-Version eher den Subskalen der Original-Publikation zugeteilt werden als die der schweizerischen TFI-Version. Es konnte wiederholt gezeigt werden, dass die Frage 22 des deutschen wie auch des schweizerischen TFI eher der Kognition als der Lebensqualität zugeordnet wurde. Auch in der Studie von Fackrell et al. [8] aus dem Vereinigten Königreich fiel auf, dass die Frage 22 neben der Lebensqualität auch die Kognition erfasst.

## Diskussion

Die Übersetzung des originalen TFI in die deutsche Sprache für Deutschland und die Schweiz erfolgte gemäß den „Principles of Good Practice for the Translation and Cultural Adaptation Process for Patient-Reported Outcomes (PRO) Measures“ der ISPOR Task Force for Translation and Cultural Adaptation [23]. In der deutschsprachigen Schweiz wird im Alltag fast nur Schweizerdeutsch, ein alemannischer Dialekt, gesprochen. Im Gegensatz dazu wird für den schriftlichen Ausdruck in der deutschsprachigen Schweiz Standarddeutsch verwendet [2]. Das Verhältnis zur Standardsprache ist für den Deutschschweizer somit ein anderes als für den Deutschen [2]. Dies führt neben den kulturellen Unterschieden zwischen Deutschland und der Schweiz zu einem anderen Sprachverständnis in den jeweiligen Ländern. Dies spiegelte sich bei der separaten Vorwärts-Rückwärts-Übersetzung der beiden Länder in unterschiedlichen deutschsprachigen TFI-Versionen wider. Anstatt zwei unterschiedliche deutschsprachige Varianten eines Fragebogens zu führen, besteht auch die Möglichkeit einer interkulturellen Adaptation eines Fragebogens, einer sogenannten Harmonisierung [1, 23]. Die Harmonisierung eines Fragebogens in einer Sprache wird gemäß „Cross-Cultural Survey Guidelines“ wenn immer möglich empfohlen, und nur wirklich notwendige Unterschiede wie beispielsweise verschiedene politische Systeme sollten in verschiedenen Fragebogenversionen einer Sprache beibehalten werden [10]. In der Schweiz sowie in Deutschland wurden jedoch die für das Land verfasste deutschsprachige TFI-Version vor einer harmonisierten TFI-Version bereits validiert [5, 18]. Aus diesem Grund schien uns eine Harmonisierung der beiden nur leicht semantisch unterschiedlichen Versionen zu einer neuen TFI Version, welche ebenfalls hätte validiert werden müssen, unverhältnismäßig. Daher war es unser Ziel, in dieser Studie einerseits die Vergleichbarkeit der beiden deutschsprachigen Versionen zu prüfen und andererseits die TFI-Versionen in beiden Sprachregionen zu evaluieren mit der Frage, ob man für die Zukunft die Verwendung einer einzigen deutschsprachigen TFI-Version empfehlen kann.

Die Populationen in Deutschland und in der Schweiz waren bezüglich des Alters und Tinnitus-Schweregrads gut miteinander vergleichbar. Die einzige Auffälligkeit war in der Geschlechterverteilung zu verzeichnen mit höherem Frauenanteil in Deutschland verglichen mit der Schweiz. Interessanterweise war die Geschlechterverteilung in Deutschland in der Studie von Brüggemann et al. [5] ebenfalls ausgeglichener bei einem Frauenanteil von 55 % im Vergleich zur Studie von Peter et al. [18] aus der Schweiz, bei welcher der Frauenanteil bei 39,4 % lag. Somit decken sich die aktuellen Daten aus dieser Studie mit denen aus den beiden anderen Studien [5, 18].

Die gesamte Population zeigte im gepaarten t‑Test zwischen den beiden deutschsprachigen TFI-Versionen keine signifikanten Unterschiede. Auch in der weiteren Aufschlüsselung bezüglich Population und Reihenfolge der TFI-Abgabe waren in den ungepaarten t‑Tests keine signifikanten Unterschiede zu verzeichnen. Im gepaarten t‑Test zeigten sich bei der weiteren Aufschlüsselung in Bezug auf Population und Reihenfolge der TFI-Abgabe einzelne signifikante Werte zwischen den beiden deutschsprachigen TFI-Versionen mit jedoch nur schwachem bis mittlerem Effekt gemäß Einteilung von Cohen [7]. Dies bedeutet, dass trotz dieser signifikanten Werte der Unterschied zwischen den beiden deutschsprachigen TFI-Versionen vermutlich nicht groß genug war, um diesen als bedeutend einzustufen. Gegebenenfalls lassen sich die teilweise erhaltenen signifikanten Werte dadurch erklären, dass die Gesamtpunktzahl höher war bei der TFI-Version, welche von den Probanden zuerst durchgeführt wurde. Da die beiden TFI-Versionen direkt hintereinander abgegeben wurden, können die Unterschiede nicht auf eine eigentliche Veränderung der Ohrgeräusch-Beeinträchtigung zurückgeführt werden. Üblicherweise besteht bei einer zu kurz gewählten Zeitspanne zwischen zwei Fragebögen die Gefahr, dass sich die Befragten an die Antworten erinnern. Bei Antwort-Möglichkeiten auf einer Likert-Skala von 0–10, wie beim TFI, scheint die Erinnerung bei 25 Fragen eher unwahrscheinlich. Die tieferen Werte bei der zweiten TFI-Version könnte daran liegen, dass beim erneuten Ausfüllen des Fragebogens bereits alle Aspekte der Ohrgeräusch-Beeinträchtigung bei der ersten Version erfragt wurden und dies zu Angabe von tieferen Werten bei der Wiederholung führte.

**Figure 2:**
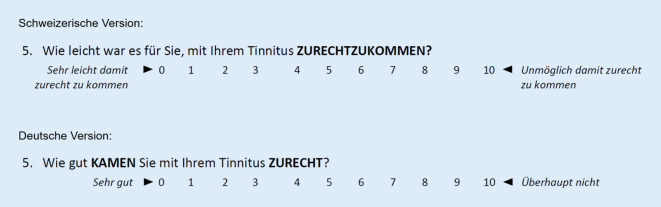

Bei signifikant unterschiedlichen Werten im gepaarten t‑Test der beiden TFI-Versionen erfolgte zusätzlich ein gepaarter t‑Test in Bezug auf die einzelnen Fragen. Die signifikant unterschiedlichen Fragen zeigten mit einer Ausnahme ebenfalls nur eine mittlere Effektstärke bei Korrelationskoeffizienten *r *unter 0,5, was als vermutlich nicht bedeutender Unterschied beurteilt werden kann. Einzig war bei der schweizerischen Population bei der Reihenfolge „schweizerische und anschließend deutsche TFI-Version“ ein Korrelationskoeffizient *r* von 0,51 bei der Frage 5 zu verzeichnen, was einem starken Effekt entspricht und somit als bedeutender Unterschied einzustufen ist. In Abb. 2 sind die beiden deutschsprachigen TFI-Versionen der Frage 5 aufgeführt. Es zeigt sich, dass nicht nur die Frage anders gestellt wurde, sondern sich auch die Skala der Antwortmöglichkeiten unterschied. Dies scheint zu diesem signifikanten Wert geführt zu haben. Bei jedoch mehrheitlich nicht signifikanten Werten im gepaarten und ungepaarten t‑Tests sowie fast ausschließlich unbedeutend signifikanten Werten in einzelnen gepaarten t‑Tests kann davon ausgegangen werden, dass die beiden deutschsprachigen TFI-Versionen gut miteinander vergleichbar sind.

Bei der Faktoranalyse mittels Jolliffe-Kriterium ließen sich in der gesamten Population bei der deutschen TFI-Version 6 Faktoren und bei der schweizerischen TFI-Version 5 Faktoren extrahieren (Tab. 1). Bemerkenswert ist jedoch, dass sich in der schweizerischen Population bei der deutschen TFI-Version ebenfalls 6 Faktoren zeigten, wohingegen bei der für die schweizerische Population verfasste schweizerischen TFI-Version nur 5 Faktoren extrahiert werden konnten.

Insbesondere der Fakt, dass die schweizerische Population in der deutschen TFI-Version besser als in seiner angedachten schweizerischen TFI-Version abschneidet, spricht dafür, dass sich die deutsche TFI-Version ohne eine interkulturelle Anpassung besser für den weiteren Gebrauch in beiden Ländern eignet. Auch die englischsprachige TFI-Version für die USA konnte in verschiedenen anderen englischsprachigen Ländern ohne interkulturelle Anpassungen validiert werden [6, 8].

## Fazit für die Praxis

- Die beiden deutschsprachigen Versionen des Tinnitus Functional Index (TFI) aus Deutschland und aus der Schweiz sind gleichwertig zu verwenden.
- Für die Zukunft wird bei leicht besseren Ergebnissen in der Faktoranalyse die Verwendung der TFI-Version für Deutschland im gesamten deutschsprachigem Raum empfohlen.

## References

[1] Aebischer B, Hill JC, Hilfiker R (2015). German translation and cross-cultural adaptation of the STarT back screening tool. PLoS ONE.

[2] Bickel H (2000). Deutsch in der Schweiz als nationale Varietät des Deutschen. Sprachreport.

[3] Biswas R, Hall DA (2020) Prevalence, incidence, and risk factors for Tinnitus. Curr Top Behav Neurosci. 10.1007/7854_2020_154 10.1007/7854_2020_154 32840860

[4] Biswas R, Lugo A, Hall DA et al (2020) A study of the pan-european prevalence of Tinnitus and hearing difficulty using a standardized set of questions. Poster presented at association of research in otolaryngology 43rd annual midwinter meeting. San Jose, California, USA. https://aro.org/wp-content/uploads/2020/02/2020-Abstracts_1-21-20-Web.pdf. Zugegriffen: 09.08.2021

[5] Bruggemann P, Szczepek AJ, Kleinjung T (2017). Validation of the German version of Tinnitus Functional Index (TFI). Laryngorhinootologie.

[6] Chandra N, Chang K, Lee A (2018). Psychometric validity, reliability, and responsiveness of the Tinnitus functional index. J Am Acad Audiol.

[7] Cohen J (1992). A power primer. Psychol Bull.

[8] Fackrell K, Hall DA, Barry JG (2016). Psychometric properties of the Tinnitus Functional Index (TFI): assessment in a UK research volunteer population. Hear Res.

[9] Goebel G, Hiller W (1994). The tinnitus questionnaire. A standard instrument for grading the degree of tinnitus. Results of a multicenter study with the tinnitus questionnaire. HNO.

[10] Harkness J, Dorer B, Mohler PP (2016) Shared language harmonization. https://ccsg.isr.umich.edu/chapters/translation/harmonization/. Zugegriffen: 11. Jan. 2021

[11] Jolliffe IT (1986). Principal component analysis.

[12] Jun HJ, Yoo IW, Hwang SJ (2015). Validation of a Korean version of the Tinnitus handicap questionnaire. Clin Exp Otorhinolaryngol.

[13] Kaiser HF (1974). An index of factorial simplicity. Psychometrika.

[14] Kamalski DM, Hoekstra CE, Van Zanten BG (2010). Measuring disease-specific health-related quality of life to evaluate treatment outcomes in tinnitus patients: a systematic review. Otolaryngol Head Neck Surg.

[15] Kleinjung T, Fischer B, Langguth B (2007). Validation of the German-version Tinnitus Handicap Inventory (THI). Psychiat Prax.

[16] Meikle MB, Henry JA, Griest SE (2012). The tinnitus functional index: development of a new clinical measure for chronic, intrusive tinnitus. Ear Hear.

[17] Newman CW, Jacobson GP, Spitzer JB (1996). Development of the Tinnitus handicap inventory. Arch Otolaryngol Head Neck Surg.

[18] Peter N, Kleinjung T, Jeker R (2017). Tinnitus functional index: validation of the German version for Switzerland. Health Qual Life Outcomes.

[19] Rabau S, Wouters K, Van De Heyning P (2014). Validation and translation of the Dutch tinnitus functional index. B-ENT.

[20] Snow JB (2016). History of the Tinnitus Research Consortium. Hear Res.

[21] Suzuki N, Oishi N, Ogawa K (2019). Validation of the Japanese version of the tinnitus functional index (TFI). Int J Audiol.

[22] Vanneste S, To WT, De Ridder D (2011). The psychometric properties of the Tinnitus handicap questionnaire in a Dutch-speaking population. Clin Otolaryngol.

[23] Wild D, Grove A, Martin M (2005). Principles of good practice for the translation and cultural adaptation process for patient-reported outcomes (PRO) measures: report of the ISPOR task force for translation and cultural adaptation. Value Health.

[24] https://apps.ohsu.edu/research/tech-portal/technology/view/1004796. Zugegriffen: 09.08.2021

